# Fluorescence changes at flow cytometric analysis of samples from sentinel lymph nodes excised with indocyanine green and methylene blue guided mapping techniques

**DOI:** 10.3389/fvets.2026.1766355

**Published:** 2026-04-02

**Authors:** Alessandra Ubiali, Elisa Maria Gariboldi, Damiano Stefanello, Roberta Ferrari, Valeria Martini

**Affiliations:** Dipartimento di Medicina Veterinaria e Scienze Animali, Università degli Studi di Milano, Lodi, Italy

**Keywords:** flow cytometry, indocyanine green, median fluorescence intensity, methylene blue, sentinel lymph node

## Abstract

**Introduction:**

Flow cytometry (FC) has been used recently to assess percentages of infiltration by mast cells in sentinel lymph nodes (SLN) of dogs with mast cell tumors (MCT). SLN mapping often includes the use of different dyes such as methylene blue (MB) and indocyanine green (ICG), which might influence fluorescence assessment by FC. This study aimed to assess whether the color given by mapping dyes might affect the baseline fluorescence of SLN aspirates for FC.

**Methods:**

Baseline fluorescence was calculated as Median Fluorescence Intensity (MFI) in 4 channels (FL1, FL2, FL3, FL4, respectively corresponding to 530/30 nm, 585/40 nm, >670 nm, 660/20 nm) with a cytometer equipped with two lasers (488 nm and 638 nm) with constant setting and compensation. SLN aspirates were suspended in RPMI medium. They were classified based on results of mapping techniques in 4 dye classes (blue/fluorescent, fluorescent/non-blue, blue/non-fluorescent, non-blue/non-fluorescent), and possible differences in MFI values among SLN dye classes were assessed.

**Results:**

Thirty-five SLNs from 17 dogs were assessed. Considering dye classes, 13 were blue/fluorescent, 16 were fluorescent/non-blue, 4 were blue/non-fluorescent, 2 were non-blue/non-fluorescent. In all fluorescence channels, except FL1, MFI varied among SLN dye classes. Blue/non-fluorescent SLNs showed the highest fluorescence, followed by blue/fluorescent, fluorescent/non-blue, and non-blue/non-fluorescent.

**Discussion:**

These results suggest that the use of dyes for SLN mapping may introduce a relevant bias when MFI is quantitatively assessed via FC.

## 1 Introduction

Flow cytometry (FC) technique is based on lasers that emit light at specific wavelengths and hit sample cells in a liquid stream. Specific staining kits or fluorochrome-labelled antibodies can be added to the cellular suspension before analysis. Once excited by the laser light, the fluorochrome emits light at a different wavelength. That light is thereafter filtered to obtain a clearer signal, recorded by specific detectors, and finally converted into numerical data by an electronic network. The higher the amount of fluorescent antibody binds to the cell, the higher the intensity of light at that specific wavelength will be produced. Finally, the numerical data obtained (Median Fluorescence Intensity, MFI) are proportional to the intensity of the light resulting from the interaction between the laser beam and the antibody-labelled cell, which in turn is proportional to the amount of protein present on the cell. Thus, besides assessing the relative prevalence of specific cellular subsets, FC plays a relevant role in assessing the degree of expression of specific molecules, enabling to move from a qualitative assessment to a quantitative one. This is commonly calculated as a ratio between the MFI obtained by antibody-labelled cells and the autofluorescence of the cells in an unstained sample (1, 2). Indeed, when cells are interrogated by the lasers, a minimal degree of light is captured by the detectors at different wavelengths even if cells were not pre-labelled with fluorescent substances, resulting in the so-called autofluorescence (3). FC has been recently proposed as a potential alternative for diagnosing nodal metastasis in dogs with mast cell tumor (MCT) (4, 5) and, even if histopathology remains the gold standard for assessing nodal metastasis in dogs, it seems plausible that FC will be further investigated in the context of sentinel lymph node (SLN) assessment in the future. To date, SLN mapping is a diffuse technique in surgical oncology of dogs, particularly for patients with MCT, and different mapping techniques have been assessed to correctly identify and extirpate the tumor-draining lymph nodes (6–10). Most of those techniques are the same of those commonly used also in human patients and include the use of specific tracers, such as Technetium-99 metastable (99mTc) for lymphoscintigraphy (8, 11–15), or dyes, such as methylene blue (MB) (8, 11) and indocyanine green (ICG) (9, 12, 14–18). In particular, 99mTc is invisible to the naked eye, while MB is detectable in the visible light spectrum (19, 20), and ICG emits in the near-infrared light spectrum (820 nm) once excited by 750-800 nm wavelength light (21, 22). Being injected peritumorally, those dyes follow the lymphatic route that connects MCT to the SLN, highlighting the latter within the sentinel lymphocentrum, ultimately resulting in an intraoperative guidance for the surgeon.

Aside from its well-recognized role in the investigation of hematological malignancies (23–25), in veterinary medicine, interest has recently extended to FC assessment of metastasis in SLN of dogs with MCT (4, 5). It seems quite straightforward that, when using FC on a sample obtained from an SLN, dyes like ICG and MB may interfere with the evaluation of autofluorescence.

Consequently, sampling an SLN that appears fluorescent or blue *in vivo* may lead to signal interference in FC, which is based on fluorescence detection.

This may occur specifically, if the emission spectrum of the dye overlaps with the fluorescence detection channels of the cytometer, or non-specifically, due to background emission within the spectral range of interest for FC (3, 26). Indeed, these molecules may exert a secondary optical effect on the detected fluorescence. Moreover, when two different dyes are used in the same patient, their interaction could potentially alter their spectral architecture, possibly leading to unexpected distortions in the emitted wavelengths.

Determining whether such alterations occur, and to what extent each fluorescence channel is affected, could provide valuable insights for the future evaluation of SLN in patients undergone to MB or ICG mapping procedures. Hence, this study aimed to assess the influence of those mapping dyes used either alone or in combination, on the autofluorescence at FC analysis of cells from ex vivo SLN aspirates of dogs with MCTs.

## 2 Methods

All samples included in this prospective study came from client-owned dogs bearing MCT that were subjected to sentinel lymphadenectomy after their owners signed a written informed consent, at the Veterinary Teaching Hospital, University of Milan, from September 2023 to December 2024. Since samples were taken ex vivo on excised lymph nodes, the study did not involve any additional procedure for tissue sampling above the planned surgical procedure; therefore, specific Ethical Committee approval to use leftover specimens for research purposes was not required (Ethical Committee decision 29 October 2012, renewed with protocol 02–2016, University of Milan).

Techniques used for mapping and detection of the SLN were: lymphoscintigraphy with 99mTc and near infrared fluorescence (NIRF) with ICG (9) with or without MB, lymphoscintigraphy with 99mTc with MB, near infrared fluorescence (NIRF) with ICG with or without MB (6, 8, 17, 27). Thus, SLN were qualitatively assessed as described in Gariboldi et al. (9) and defined as follows: blue/non-blue according to the direct visualization of the SLN and fluorescent/non-fluorescent based on intraoperative assessment using a specific portable handheld camera for NIRF detection (SPY-PHI, Stryker, MIDA, Tecnologia Medica S.p.a). SLNs that were non blue and positive only at lymphoscintigraphy assessment using a handheld intraoperative gamma-probe (HIGP–Crystal probe SG04; Crystal Photonic GmbH, Berlin, Germany), either because ICG was not used, or because NIRF-ICG assessment showed absence of fluorescence, were classified as non-blue/non-fluorescent.

Finally, SLNs were divided into four dye-classes based on mapping results: blue/fluorescent, fluorescent/non-blue, blue/non-fluorescent, and non-blue/non-fluorescent.

All extirpated SLNs were immediately sampled via fine needle aspiration (FNA) in the operating room, irrespective of the dye class assigned. The material was suspended in RPMI medium and processed for FC within 24 h from collection. Both the primary lesion and the extirpated SLN(s) were then subject to histopathological assessment for diagnostic and staging purposes.

**Figure 1:**
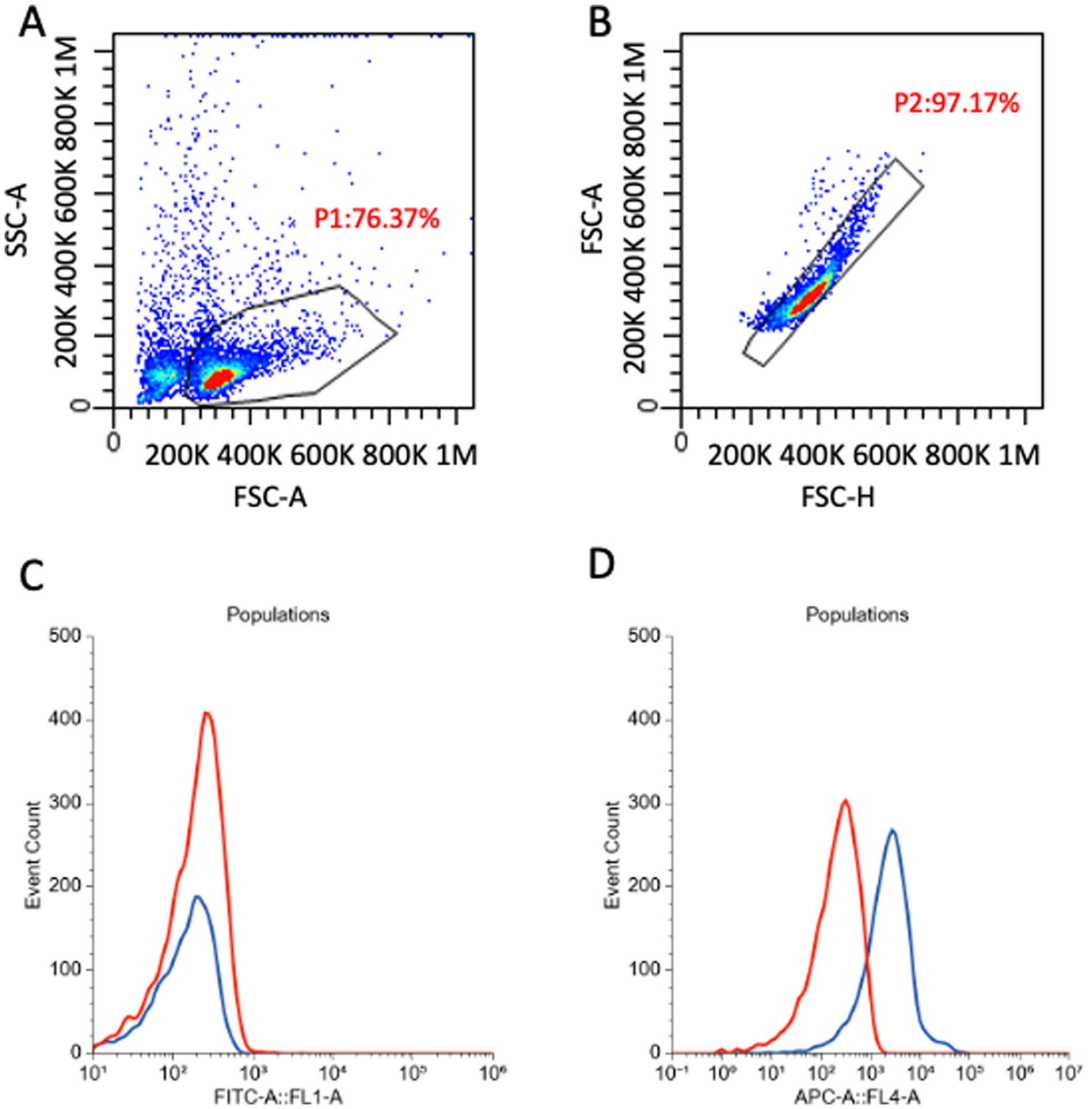
Gating strategy to assess autofluorescence of cells in 35 nodal samples collected *ex vivo* after *in vivo* mapping with different dyes. **(A)** Density plot, all events are shown. A first gate (P1) is set to select only the lymphocyte population, based on morphological characteristics. **(B)** Density plot, P1 events are shown. A gate (P2) was set to exclude doublets. **(C,D)** Histogram overlay showing, respectively, FL1 (530/30 nm) **(C)** and FL4 (660/20 nm) **(D)** fluorescence of two representative samples. Red line, non-blue SLN; blue-line, blue SLN. The two lines overlap when considering FL1, where the fluorescence is similar for different *in vivo* stained SLNs, while in FL4 the blue SLN shows higher fluorescence than the non-blue one. **(C,D)** were obtained with the free online software floreada.io.

For FC assessment, samples were processed according to the standard procedures of the laboratory, which have already been described in the literature (28). Briefly, an automated cell count was performed, and different sample volumes were placed in a tube containing a final count of 500,000 cells. Subsequently, 25 μL of a blocking solution containing 10% fetal bovine serum (FBS) and 0.2% sodium azide in RPMI was added. The tubes were incubated for 10 min, and then 1 mL of an RBC lysis buffer was added. Samples were centrifuged at 1500 rpm for 8 min; the supernatant was removed, and cell pellets were resuspended in 500 μL of PBS. Further tubes were prepared in the same way, and incubated with different antibody cocktails, for the purposes of another concomitant project. Acquisition was made with a flow cytometer (BriCyte E6, Mindray, Shenzen, China) which was equipped with 2 lasers (488 nm and 638 nm) and had 4 fluorescence channels (FL1, FL2, FL3, FL4, respectively, corresponding to 530/30 nm, 585/40 nm, >670 nm, 660/20 nm). At the beginning of each laboratory session, a quality control check was performed with cytometer-specific controls (Rainbow Calibration Particles, Mindray). Instrument setting and compensation matrix were then kept constant. Specifically, the compensation matrix was generated using cells from nodal aspirates routinely processed in our laboratory for diagnostic purposes. All scattergrams were visually inspected during acquisition and analysis, and no evidence of over- or under-compensation was observed. Data were analyzed with specific software “MRflow” (Mindray) by a single experienced operator (VM), who was blind to the mapping procedure used and to the dye class of each specific SLN. A gate was set to include only lymphoid cells and exclude platelets and debris. A second gate was set to exclude doublets. Finally, fluorescence data of unstained samples were recorded and reported as MFI for each fluorescence channel (Figure 1).

### 2.1 Statistical analyses

Descriptive statistics were calculated. Data from continuous variables were reported as median (1st and 3rd quartile), to ensure consistency and robustness of their presentation.

Statistical differences in MFI values of unstained samples among the four SLN dye classes were assessed with Generalized Estimating Equations (GEE) for gamma distribution with log link and sandwich standard errors. The choice of GEE was based on the observation that 9 dogs contributed to the study with multiple samples (from 2 to 5 samples each). Thus, considering the correlation among samples coming from the same patient, and the unequal cluster sizes, case ID was considered as a clustering unit for the analysis, with an exchangeable correlation structure. Models were fit for fluorescence channel.

All analyses were performed with SPSS v 29.0 for Windows, and significance was set at *p* ≤ 0.05 for all tests.

## 3 Results

A total of 17 dogs were included. Based on tracers availability, in order to ensure a better identification, detection and removal of SLN, the mapping technique used was a combination of NIRF-ICG plus MB in 7 dogs, NIRF-ICG plus lymphoscintigraphy in 4, NIRF-ICG plus lymphoscintigraphy plus MB in 3, in 2 lymphoscintigraphy plus MB, and lastly, in one dog, NIRF-ICG only was used. A total of 35 SLNs were excised and included for FC evaluation. The subdivision in the 4 dye classes is reported in Table 1.

**Table 1:** MFI values of the four fluorescence channels based on SLN dye classes.

SLN dye class	*n* (%) total = 35	MFI fluorescence channel median (IQR)			
		FL1	FL2	FL3	FL4
Blue/fluorescent	13 (37%)	152.5 (122.0–215.0)	72.0 (56.5–128.0)	265.0 (96.5–686.0)	301.0 (59.0–751.5)
Fluorescent/non-blue	16 (46%)	156.5 (137.3–179.8)	63.5 (54.3–80.5)	119.5 (93.9–161.3)	40.0(23.5–97.0)
Blue/non-fluorescent	4 (11%)	245.5 (106.3–391.5)	196.5 (58.0–376.3)	1426.0 (411.8–4473.5)	2394.0 (805.3–12402.3)
Non-blue/non-fluorescent	2 (6%)	116.0; 335.0	5.0; 217.0	175.0; 400.5	50.5; 220.0

Notes: SLN, sentinel lymph node; MFI, median fluorescence intensity; IQR, interquartile range; FL, fluorescence channel.

Overall, median MFI of unstained samples was 154 (range, 100–394) for FL1, 67 (range, 42–392) for FL2, 175 (range, 51–5278) for FL3, and 79 (range, 11.9–15,575) for FL4.

**Figure 2:**
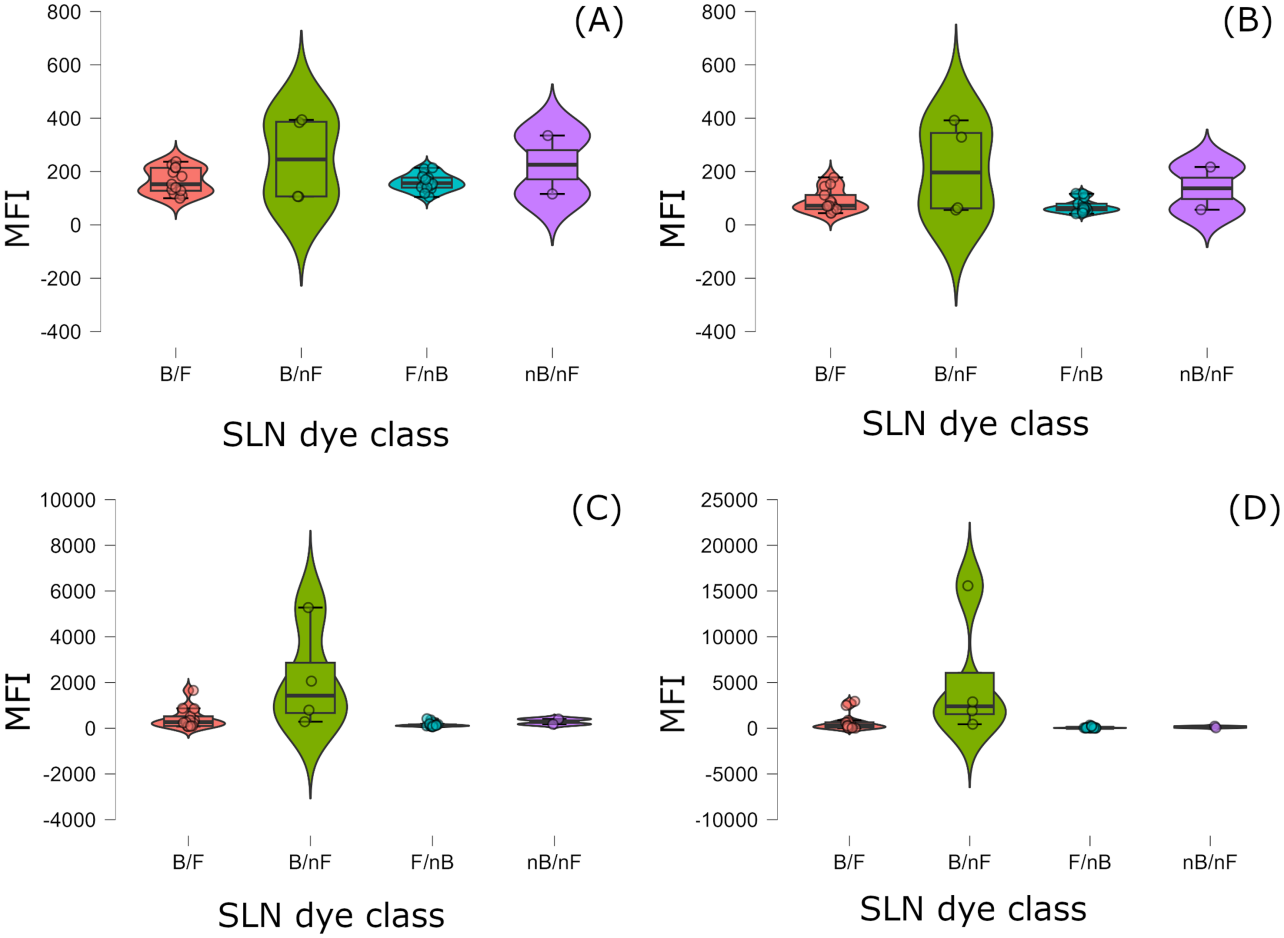
Violin plots showing per-sample distributions of MFI across the SLN dye classes. **(A)** FL1; **(B)** FL2; **(C)** FL3; **(D)** FL4. FL, fluorescence channel; MFI, median fluorescence intensity; SLN, sentinel lymph node; B/F, Blue/Fluorescent; B/nF, Blue/non-fluorescent; F/nB, Fluorescent/non-blue; nB/nF, non-blue/non-fluorescent.

Median (1st and 3rd quartile) of samples for each dye class are reported in Table 1 and depicted in Figure 2. A significant difference in MFI values among SLN dye classes was detected for FL2 (*p* = 0.002), FL3 (*p* < 0.001) and FL4 (p < 0.001), but not for FL1 (*p* = 0.375). In particular, blue/non-fluorescent SLN had the highest MFI values in all channels, blue/fluorescent SLN had intermediate values, whereas non-blue/non-fluorescent and non-blue/fluorescent SLN had overlapping, low values.

Despite differences in autofluorescence among classes, this variation did not impair lymphocyte population detection, which was always clearly defined (Supplementary Figure 1).

## 4 Discussion

In the present paper we reported a significant interference in the fluorescence intensity at three different wavelengths when either a single dye or a combination of MC and ICG was used for sentinel lymph node mapping and excision.

Each dye can emit light at specific wavelengths causing varying degrees of signal interference. Specifically, ICG is a fluorophore excited at 780–805 nm and emits at 820–840 nm (29). In our study, the lasers used operated at 488 and 638 nm. Thus, we did not expect significant interference from ICG. Conversely, MB, in addition to emitting within the visible light spectrum, has been reported to show an emission peak at 688 nm (30), which may partially overlap with the wavelengths detected in the present study (mainly FL3 and FL4, that are commonly used for PerCP, and APC/AF647 fluorochromes, respectively).

Our results demonstrated different prevalence of samples with high MFI values in FL2, FL3, and FL4 among blue/fluorescent, fluorescent/non-blue, blue/non-fluorescent, non-blue/non-fluorescent SLNs, leading to significant statistical differences. Conversely, higher overlap in MFI was found for FL1 channel, leading to lack of statistical differences (Table 1 and Figure 2). This result was partially expected, since the range of detection of FL1 (530/30 nm) lies far from the emission peaks of both MB and ICG. Still, minimal influence on autofluorescence even in the FL1 channel was present.

Regarding other fluorescence channels, blue/non-fluorescent SLNs showed the highest fluorescence, followed by blue/fluorescent and fluorescent/non-blue and non-blue/non-fluorescent ones. This observation is consistent with the fact that MB is the only molecule reported to emit effectively within the wavelength range detected by our cytometer. Conversely, at FC assessment, ICG produced only a minor autofluorescence.

Interestingly, SLNs that were blue/fluorescent exhibited intermediate MFI values between blue/non-fluorescent and fluorescent/non-blue SLNs. This finding could be explained by possible alterations in the optical properties of MB and ICG when combined together, potentially due to molecular proximity or non-covalent interactions, anyway to the author knowledge no studies focused on this specific property.

Overall, our findings suggest that variations in the level of autofluorescence of the cells may introduce a relevant bias when MFI is quantitatively assessed in SLN from dogs that received either dye for SLN mapping. It could be speculated that nodal sampling before administration of any dye could help overcome this problem. We do not support such an approach, since the identification of the specific sentinel lymphocentrum and SLN within a lymphocentrum is only possible if using a mapping technique (8, 11, 17). Thus, sampling prior to the administration of any dye could lead to misidentification of the SLN. Awareness of the interference of each dye on FC results can help interpreting results from this technique, avoiding erroneous sampling of non-sentinel lymph nodes.

Furthermore, it is noteworthy to underlie that the influence on the autofluorescence of the cells did not prevent us to detect lymphocytes populations (Supplementary Figure 1), therefore the impact of intraoperative dyes has to be taken into account in a contest in which a quantitative assessment of the fluorescence of the sample is considered, and in that case, would be suggested to prefer SLNs detected by non-visible dyes such as lymphoscintigraphy with 99mTc, or to limit the analysis to the less affected fluorescence channel (FL1, 530/30 nm).

Considering that the injected dye typically follows the lymphatic flow to the lymph node, it is possible that part of it remains in the extracellular matrix while another portion is taken up by the cells. Therefore, performing multiple washes of the sample may help remove any unbound or freely diffusing dye, thereby reducing background fluorescence. This was not performed in the present study, since we aimed to mimic the standard operative procedures commonly used in veterinary FC facilities.

Anyway, the advantage of additional washing steps should be carefully evaluated and balanced with possible disadvantages, since it would result in additional sample handling, and, based on the authors’ experience, it could cause cell loss and delay in sample acquisition and analysis.

The main limitations of the present study are related to its exploratory nature, mainly regarding the number of samples collected for each mapping technique and the inclusion of SLNs extirpated with different combinations of them. Most importantly, only 2 non-blue/non-fluorescent samples were included. This could have impacted on the statistical results of our study. Still, those two samples represented the true paragon of all other dye classes, since their MFI was linked to cellular autofluorescence, without uptake of any kind of dye.

In particular, either methylene blue or NIRF-ICG are often used in combination of other techniques as they help the surgeon to visualize SLN during extirpation, showing very good performances, with a really low number of SLN without blue or fluorescence uptake reported to date (9, 27, 31). The low number of non-fluorescent/non-blue SLN in the present study clearly reflects this tendence, but created an important limitation leading to a low number of samples for this category. In the future, to confirm this data, more samples have to be included. In particular, expansion of the non-blue/non-fluorescent and blue-only groups would be necessary to strengthen statistical comparisons. Since sampling was performed in a clinical setting, and although all patients received the same peritumoral dose of tracer, the actual concentration, dilution, and distribution of dyes were beyond our control and could not be measured.

Consequently, part of our results may have been influenced by patient-specific characteristics of drainage, leading to different uptake of the dyes by SLNs overall from one dog to another. Anyway, this should have been partially prevented by use of GEE test rather than Kruskal-Wallis one, which would not have kept into consideration possible correlation among samples. Despite this, further variables that were not considered in the present study could possibly have biased the results, including timing from dye injection to sampling and processing for FC, as well as breed peculiarities or differences among anatomical location of the lymphocentrum involved, tumor cells burden and type of cells sampled (32–34), cell viability (35–37), and others. Those same variables could have led to the high intra-dye class variability observed in the present study. All of them need to be addressed in future studies.

## 5 Conclusion

ICG and MB, when used for SLN mapping, may influence the baseline fluorescence of cells in nodal aspirates during FC analysis. Specifically, MB has a stronger influence than ICG or their combination, with FL2, FL3 and FL4 channels being more affected than FL1. This potential interference should be taken into account when performing quantitative fluorescence assessments on SLN samples obtained with mapping with MB and/or ICG in future studies.

## Data Availability

The raw data supporting the conclusions of this article will be made available by the authors, without undue reservation.

## Supplementary material

Supplementary Figure 1 Example of a sample from a blue SLN that was stained with CD21-AF647 and CD45-FITC **(A)**, CD5-PerCP-EF710 and CD45-FITC **(B)**, CD4-FITC, and CD8-PE **(C)** antibodies to assess lymphocytes subpopulations. The populations are clearly identifiable and not affected by the baseline fluorescence due to the *in vivo* staining of the SLN.
