# Supplementary figures and images for: Fluorescence changes at flow cytometric analysis of samples from sentinel lymph nodes excised with indocyanine green and methylene blue guided mapping techniques
Source: Front Vet Sci. 2026 Apr 2;13:1766355. doi: 10.3389/fvets.2026.1766355 (PMC13085631; doi:10.3389/fvets.2026.1766355)

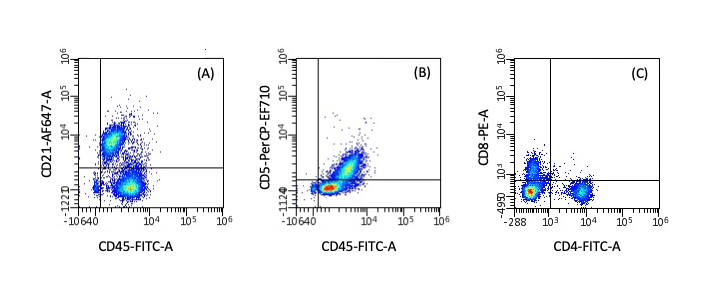

Supplement: Supplementary Figure 1 — Example of a sample from a blue SLN that was stained with CD21-AF647 and CD45-FITC (A), CD5-PerCP-EF710 and CD45-FITC (B), CD4-FITC, and CD8-PE (C) antibodies to assess lymphocytes subpopulations. The populations are clearly identifiable and not affected by the baseline fluorescence due to the in vivo staining of the SLN. [file Image_1.tif]
